# Dr. D. Eugene Block

**Published:** 1917-01

**Authors:** 


**Dr. D. Eugene Block,** Toronto University, Veterinary Dept. died of typhoid fever in Lowell, Mass., Dec. 16. He was born in Buffalo May 22, 1873, was educated in the public schools of this city and practiced here for a number of years, moving to Lowell about three years ago. The funeral was held from the residence of his sister, Mrs. Nathan Desbecker, of Buffalo.

